# Complete Genome Sequence of *Myxococcus stipitatus* Strain DSM 14675, a Fruiting Myxobacterium

**DOI:** 10.1128/genomeA.00100-13

**Published:** 2013-03-21

**Authors:** Stuart Huntley, Susanne Kneip, Anke Treuner-Lange, Lotte Søgaard-Andersen

**Affiliations:** Max Planck Institute for Terrestrial Microbiology, Marburg, Germany

## Abstract

Hallmarks of the myxobacteria include the formation of spore-filled fruiting bodies in response to starvation and synthesis of secondary metabolites. *Myxococcus stipitatus* forms morphologically highly distinct fruiting bodies and produces secondary metabolites with antibiotic or cytotoxic activities. Here, we present the 10.35-Mb genome sequence of *M. stipitatus* strain DSM 14675.

## GENOME ANNOUNCEMENT

Most myxobacteria initiate a complex developmental program in response to nutrient starvation (1). The end result of this program is the formation of fruiting bodies that are filled with environmentally resistant myxospores. Fruiting body morphology is genetically determined and varies widely among myxobacterial species, from simple spore-filled masses to complex tree-like structures (1, 2). Among myxobacteria, *Myxococcus xanthus*, which belongs to the suborder *Cystobacterineae*, has emerged as a model organism to understand fruiting body formation (3). Within the genus *Myxococcus*, most members generate simple haystack-shaped fruiting bodies. However, *M. stipitatus* generates a more complex fruiting body structure in which a mass of myxospores is placed on top of a cell-free stalk (2). Myxobacteria are also rich sources of secondary metabolites, several of which have antibiotic or cytotoxic activities (4, 5). Among secondary metabolites produced by *M. stipitatus*, melithiazols have been shown to have antibiotic activity (6) and rhizopodin has been shown to have cytotoxic activity (7). However, the gene clusters encoding the enzymes involved in their biosynthesis have not been identified.

As a part of our ongoing efforts to understand the evolution of the genetic programs for fruiting body formation as well as the genetic basis for differences in fruiting body morphology within the myxobacteria, we sequenced and annotated the entire genome of the proposed neotype strain *M. stipitatus* DSM 14675 (8), which was obtained from the Deutsche Sammlung von Mikroorganismen and Zellkulturen GmbH (DSMZ). After verifying fruiting body formation, we collected genomic DNA and sequenced it using the 454 XLR Titanium platform on an 8-kb paired-end library and Illumina genome analyzer IIx 100-bp reads. A combined total of 8,941,970 filtered reads (466,513 Titanium and 8,475,457 Illumina reads; 102-fold coverage) were assembled into a single scaffold using Newbler (9) and Celera (10) assembler results. Sanger-based sequencing was performed to close remaining gaps and to verify the assembly. Genome annotation was prepared by manual curation of the combined predictions of RAST (11) and PRODIGAL (12) algorithms.

The complete sequence of the *M. stipitatus* DSM 14675 single chromosome genome contains 10,350,586 bp, with a G+C content of 69.2%. Seventy-six tRNA genes and three rRNA operons were identified, along with 8,043 protein-coding genes, which average 1,175 bp in length. Predicted genes total 91.5% of the entire genome sequence. The size and genetic content of the *M. stipitatus* genome are similar to those of the other completely sequenced genomes of fruiting myxobacteria, i.e., *Sorangium cellulosum* of the suborder *Sorangineae* (13) and *Haliangium ochraceum* of the suborder *Nannocystineae* (14), as well as *M. xanthus* (15), *M. fulvus* (16), *Stigmatella aurantiaca* (17), and *Corallococcus coralloides* (18) of the suborder *Cystobacterineae*, with genome sizes of 13.0 Mb, 9.4 Mb, 9.1 Mb, 9.0 Mb, 10.3 Mb, and 10.0 Mb, respectively. When visualized using the Gepard dotplot generator (19), the overall synteny of the *M. stipitatus* genome most closely matches that of *M. xanthus* and shows a single large inversion compared to that of *M. fulvus*.

### Nucleotide sequence accession number. 

The genome sequence was deposited in GenBank under accession number CP004025.
